# Reinforcement Learning Based Topology Control for UAV Networks

**DOI:** 10.3390/s23020921

**Published:** 2023-01-13

**Authors:** Taehoon Yoo, Sangmin Lee, Kyeonghyun Yoo, Hwangnam Kim

**Affiliations:** School of Electrical Engineering, Korea University, Seoul 02841, Republic of Korea

**Keywords:** wireless network topology, UAV formation control, deep reinforcement learning, step optimization

## Abstract

The recent development of unmanned aerial vehicle (UAV) technology has shown the possibility of using UAVs in many research and industrial fields. One of them is for UAVs moving in swarms to provide wireless networks in environments where there is no network infrastructure. Although this method has the advantage of being able to provide a network quickly and at a low cost, it may cause scalability problems in multi-hop connectivity and UAV control when trying to cover a large area. Therefore, as more UAVs are used to form drone networks, the problem of efficiently controlling the network topology must be solved. To solve this problem, we propose a topology control system for drone networks, which analyzes relative positions among UAVs within a swarm, then optimizes connectivity among them in perspective of both interference and energy consumption, and finally reshapes a logical structure of drone networks by choosing neighbors per UAV and mapping data flows over them. The most important function in the scheme is the connectivity optimization because it should be adaptively conducted according to the dynamically changing complex network conditions, which includes network characteristics such as user density and UAV characteristics such as power consumption. Since neither a simple mathematical framework nor a network simulation tool for optimization can be a solution, we need to resort to reinforcement learning, specifically DDPG, with which each UAV can adjust its connectivity to other drones. In addition, the proposed system minimizes the learning time by flexibly changing the number of steps used for parameter learning according to the deployment of new UAVs. The performance of the proposed system was verified through simulation experiments and theoretical analysis on various topologies consisting of multiple UAVs.

## 1. Introduction

Recent advances in Unmanned Aerial Vehicle (UAV) technology have opened up the possibility of utilizing UAVs in many research and industrial areas. Initially, UAVs were developed for military purposes, but now UAVs can be used in a wide variety of civilian fields, such as video shooting, surveillance for crime investigations, logistics delivery, and communication infrastructure provision [1]. Meanwhile, UAVs have also become smaller, cheaper to use, and have advanced in terms of freedom and maneuverability. This has led to the emergence of approaches to flocking multiple UAVs for different missions. As a representative example of such an approach, there are many studies to provide a network infrastructure using UAVs in an area where there is no infrastructure or in a disaster situation in which the network infrastructure is lost. In such a network environment, using the high mobility and freedom of UAVs to the fullest and using multiple UAVs in a swarm has the great advantage of quickly providing a low-cost network.

However, in order to provide an efficient network topology by utilizing these multiple UAVs, it is necessary to provide an optimal network topology in consideration of network operating conditions according to the constantly changing surrounding environment. In other words, the network topology must be structured in a way that maximizes network throughput and energy efficiency while maintaining connectivity between UAVs. In a typical conventional UAV network control, each UAV makes a link connection to all UAVs within transmission range. Numerous link connections can provide stability to the entire network, but it is extremely inefficient. Those link connections generate more network overhead and consume more power than is actually required.

Although some research has been conducted to control the transmission power of a network topology composed of numerous nodes [1], the presence of a centralized controller may limit the scalability of network control using multiple UAVs. To overcome these limitations, a control system that configures a network topology for multiple UAVs based on the Minimum Spanning Tree (MST) concept can be a desirable solution. With this scheme, each UAV partitions its surrounding 3D space into a given number of subspaces, then builds up an MST for each subspace, and finally it can create a forest of multiple MSTs that forms a topological network structure that minimizes power consumption, maximizes network throughput, and maintains optimal connectivity for a given formation of UAVs. However, there are two important issues to address in this system. The one is how to optimally determine the number of subspaces, since each UAV has its own connectivity and network environment that varies over time; the other one is to speed up the procedure of deciding that number. To address the first issue, we employ reinforcement learning, specifically deep deterministic policy gradient (DDPG). The resulting scheme allows the network topology to efficiently learn to maximize energy efficiency and network throughput in a continuous action space. Then, to resolve the second issue, the proposed scheme speeds up the learning speed by flexibly changing the number of steps to adjust parameter learning whenever a new UAV is deployed. Finally, the resulting network adapts to a given environment and provides an optimal network topology, providing a drone network with optimal energy efficiency and network throughput at high speed. To summarize the contributions of this paper:

- The proposed system can provide an optimal network topology by using multiple UAVs in an environment without network infrastructure.
- The proposed system can construct a UAV network topology network by leveraging MST, which can optimize energy consumption and network throughput.
- The proposed system learns the optimal network topology using RL.
- The proposed system adjusts the number of steps used in RL to reduce training time for parameters and optimize learning.

The remainder of this paper is organized as follows. Section 2 introduces prior research on UAV networking technology, RL, and ADAM optimizer. Section 3 describes the overall design of the proposed system. Section 4 shows the experiments and performance evaluation results. Finally, Section 5 concludes this paper with explaining remarks.

## 2. Related Work

Many other studies are underway related to our work to build a wireless network topology utilizing multiple UAVs. This section describes recent studies related to UAV formation algorithm, DDPG algorithm of RL, and ADAM Optimizer.

Research on the problem of arranging multiple UAVs in optimal positions is being conducted from various aspects. Depending on the purpose, multiple UAVs can perform various missions such as obstacle collision avoidance [2], flight information of multiple UAVs [3], and surveillance tasks such as target reconnaissance [4]. For the successful use of UAVs in a variety of missions, there have been studies using a deep learning approach to accurate localization techniques to improve accuracy [5] or using ultra-wideband positioning system to provide high positioning accuracy and low latency for indoor environments [6]. In addition, in order to improve the accuracy of UAV position estimation, a study using RTCM messages of RTK-GPS was conducted [7]. Furthermore, in relation to the problem of transmitting information distributed in the phased array of several UAVs, a study was conducted to formulate it as a differential game problem [8]. They tried to solve this by designing an open-loop Nash strategy rather than a classical Nash strategy. Since the algorithm for forming such a UAV aims to construct an optimal network topology according to a given mission performance environment, it is possible to construct an optimal UAV network topology through a system that can control the dynamic network topology.

Research on the problem of deploying multiple UAVs in optimal locations is closely related to the problem of constructing optimal wireless network topologies. Since the key parameters considered in the UAV positioning problem are also used as key parameters in the network topology configuration, a series of studies related to UAV position have a direct impact on the network topology configuration. There is also an approach to achieve energy efficiency by optimizing fuel consumption in practical UAV operation [9]. There are also attempts to solve the energy efficiency problem by utilizing genetic algorithms when constructing a large number of UAVs [10]. An approach to construct an optimal network topology by applying game theory to the problem of energy efficiency [11], or a study to solve the problem of energy efficiency by efficiently managing the collected data in terms of energy is in progress [12]. In this paper, the problem of energy efficiency is solved through efficient configuration of network topology using MST [13,14].

The DDPG algorithm is an algorithm that extends the idea of deep Q-learning into continuous action domains [15]. Learning in these continuous action domains can be applied in a variety of fields. Even if sparse rewards are provided, DDPG can be quickly learned when implemented in real world environments such as robots [16]. Effective results can be obtained because learning proceeds with continuous action domains even in complex environments where cars are trained for autonomous driving [17]. DDPG can also be used to find optimal voltage control in operating power grids [18]. As follows, DDPG shows good performance in various learning in continuous action space. Consequently, it can also be applied to the problem of partitioning a three-dimensional continuous space for constructing a network topology.

ADAM is a method for optimizing parameter learning in reinforcement learning by referencing past gradient changes and applying variable step sizes based on them. ADAM is known to have superior performance compared to other optimizers. ADAM has already shown optimal performance in the field of RL, but various studies are being conducted to develop it [19,20]. As one of the various studies, there have been attempts to converge faster using the Nesterov method than using the momentum method when using exponential moving averages [21]. In another paper, research on the problem of convergence to local minima that may occur while using ADAM is conducted. The authors solve the problem by using an exponential moving average that gives small weights to historical information [22]. In addition, studies have been conducted to prove the convergence of the ADAM optimization method [23].

## 3. System Design

### 3.1. System Concept

**Figure 1:**
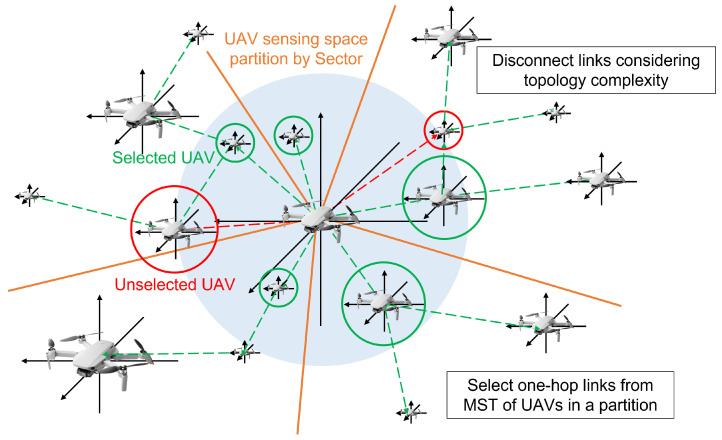
The overview of proposed topology control.

We propose a RL-based UAV network topology control system that optimizes a network consisting of multiple UAVs moving in swarms. The proposed system aims to continuously maintain an optimal network topology by reflecting changes in the position information of each UAV. Figure 1 shows the overview of the proposed system. UAVs deployed in three-dimensional space construct a network topology based on space partitioning technology. In the next step, RL is used to reflect the real-time position information of UAVs and provide an optimal network topology. Finally, we optimize the entire system by shortening the learning time of RL by adjusting the size and number of step functions.

**Figure 2:**
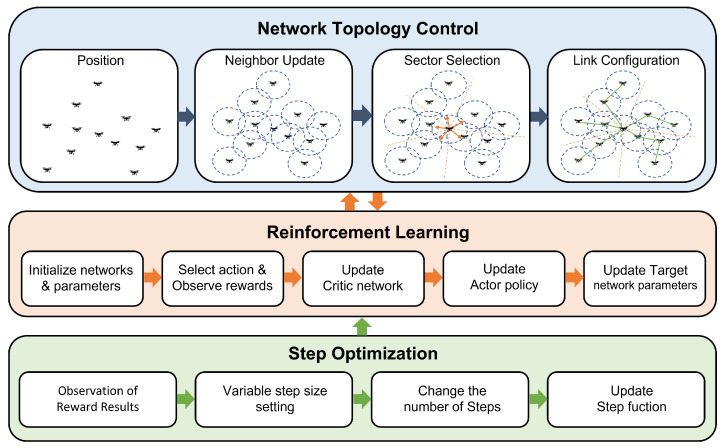
The overall oprations of proposed system.

Figure 2 shows the overall learning procedures in the system. The proposed system consists of three modules: network topology control, RL and step optimization. Through the network topology module, each UAV collects connection information for neighboring UAVs within its maximum transmission range. Each UAV then partitions the surrounding 3D space into multiple subspaces according to a given number of subspaces, and then builds a minimal spanning tree (MST) based topology for each subspace. Finally, these topologies reshape the connections, preserving the original connections obtained when gathering information from neighboring UAVs. Therefore, the problem of how many spaces to divide for each UAV is fundamental in the proposed network topology module.

In the RL module, the optimal network topology is learned through the actor and critic network of the neural network and policy updates. The reward calculated in this process updates the parameters of the critic network, actor policy, and target network. Finally, the Step Optimization module optimizes the step function used for RL to reduce the training time. This module observes the reward values derived as a result of learning and continuously adjusts the size and number of training steps. Using variable training steps reduces the training time for real-time changing network topologies and increases the adaptability of the entire system.

### 3.2. Network Topology Control

This subsection describes the UAV network topology control module. Each UAV recognizes other UAVs within the maximum transmission range. Next, the UAV divides the space around itself according to the number of subspaces. Then, it connects the links by selecting the nearest other UAVs in the divided spaces. Consequently, the network topology control algorithm constructs an optimal network topology using MST for each patitioned space. Table 1 lists the variables used in proposed system and Algorithm 1 shows the pseudocode for network topology control algorithm.
**Algorithm 1** Network Topology Control Algorithm1:Initialize neighbor UAVs’ position, distance, geometry vector:$P_{UAV},D_{UAV},G_{UAV}\leftarrow \emptyset$2:**for** $1<episode<Epi_{max}$ **do**3:   Update neighbor UAVs’ position: $P_{UAV,t}\leftarrow$ updated position4:   Calculate Distance between UAVs: $D_{UAV,t}\leftarrow$ calculated distance5:   Select links from the MST of UAVs in a partition:    $G_{UAV,t}\leftarrow$ updated link information6:   Get average hop-count, power, and degree information related to network topology:   $T_{hop,t},T_{power,t},T_{degree,t}\leftarrow G_{UAV,t}$7:   Set state from hop-count, power, and degree information:   $S_{TOPO,t}=\left[T_{hop,t},T_{power,t},T_{degree,t}\right]$8:**end for**

Algorithm 1 is performed to construct a network topology of UAVs. At the beginning of the algorithm, the current position information of the UAVs is updated in $P_{UAV}$. Beacon messages are used when collecting location information. Each UAV periodically broadcasts a beacon message specifying its current location, disseminating its location information throughout the network in real time. Next, distance information $D_{UAV}$ to other surrounding UAVs is calculated based on the updated position information of UAVs. The network topology control module connects the UAVs with the calculated distance information of the UAVs. Periodically, each UAV divides its transmission range evenly into several partitions. In each partitioned space, the UAV selects one or more nearby UAVs and removes other links. This process removes unnecessary link connections and configures an MST-based network structure in the divided subspace.

Since each UAV determines the number of links to be connected with nearby UAVs based on the number of divided partitions, reasonable space partitioning is essential for optimal link connectivity. If the number of partitions is large, too many link connections will be formed over the whole network and power consumption will increase. If the number of partitions is small, too small link connections can generate a lot of network overhead. As a result, we introduce the concept of reinforcement learning for optimal space segmentation, which is discussed in a subsequent section. The completed network topology is stored in $G_{UAV}$ and used for evaluation of the entire network topology.

### 3.3. Reinforcement Learning

In configuring a network topology using multiple UAVs, the problem of determining the number of sectors to partition is an important problem for efficient network topology control. According to Algorithm 1, each UAV builds an MST-based network topology for each subspace, which may be optimal for that space. However, it may not be optimal over the entire space, as it cannot be assumed that the UAV placement follows a uniform distribution in terms of number and location of UAVs. Thus, the process of determining how each UAV partitions the space around it (or into how many subspaces) determines the final optimality of the overall network topology. We adopt the RL to optimize sector partitioning problems.

In order to train the proposed system, we had to find a suitable algorithm for learning continuous changes in the 3D space. Compared with Deep Q-Networks (DQN), the DDPG is known to continuously expand the action space to enable a variety of learning in more dimensions. The DDPG is an off-policy algorithm that combines the advantages of DQN with an actor-critic approach, and is a policy gradient algorithm that computes deterministic policies. DDPG uses a replay buffer to reduce correlation between learning samples. Since the proposed system needs to use a continuous action space for spatial division in 3D environment, the DDPG that learns in a continuous space is suitable.

Algorithm 2 shows the detailed process of RL of the proposed system. In the training of the system, the agent observes the state $S_{t}$ of the environment at time step *t*. The actor network $Q(S,A|\theta^{Q})$ with the network parameter $\theta^{Q}$ is used to determine the action to perform in a given state $S_{t}$. The agent performs the selected task, the state transitions to the new state $S_{t+1}$, and the agent receives the reward $R_{t}$. For every time step, the agent stores the trajectory segments $\left[S_{t},A_{t},R_{t},S_{t+1}\right]$ in the replay buffer. A critic network $\mu (S|\theta^{\mu })$ with network parameter $\theta^{\mu }$ evaluates whether the behavior led the agent to a better state, and the feedback from the critic network is used to optimize the actor network. Based on the DDPG, after a set number of steps, a gradient for optimization of the actor and critic networks is calculated using the replay buffer.
**Algorithm 2** RL Algorithm1:Randomly initialize critic network $Q(S_{TOPO},A_{TOPO}|\theta^{Q})$ and actor $\mu (S_{TOPO}|\theta^{\mu })$ with weights $\theta^{Q}$ and $\theta^{\mu }$2:Initialize target network $Q^{\prime }$ and $\mu^{\prime }$ with weights $\theta^{Q^{\prime }}\leftarrow \theta^{Q}$, $\theta^{\mu^{\prime }}\leftarrow \theta^{\mu }$3:Initialize replay buffer *B*4:**for** $1<episode<Epi_{max}$ **do**5:   Initialize a random process *N* for action exploration6:   Receive initial observation state $S_{1}$7:   **for** $1<t<Step_{max}$ **do**8:     Select action $A_{t}=\mu \left(S|\theta^{\mu }\right)+N_{t}$ according to the current policy and exploration noise9:     Execute action $A_{TOPO,t}$ and observe reward $R_{TOPO,t}$ and observe new state $S_{TOPO,t+1}$10:     Store transition $\left(S_{TOPO,t},A_{TOPO,t},R_{TOPO,t},S_{TOPO,t+1}\right)$ in *B*11:     Sample a random minibatch of *N* transitions $\left(S_{t},A_{t},R_{t},S_{t+1}\right)$ from *B*12:     Set $y_{TOPO,t}=R_{TOPO,t}+\gamma Q^{\prime }\left(S_{TOPO,t+1},\mu^{\prime }\left(S_{TOPO,t+1}|\theta^{\mu^{\prime }}\right)|\theta^{Q^{\prime }}\right)$13:     Update critic by minimizing the loss : $L_{TOPO}=\frac{1}{N}\sum_{t}^{}{\left(y_{TOPO,t}-Q\left(S_{TOPO,t},A_{TOPO,t}|\theta^{Q}\right)\right)}^{2}$14:     Update the actor policy using the sampled policy gradient : $\nabla_{\theta^{\mu }}J\approx \frac{1}{N}\sum_{t}^{}\nabla_{A}Q\left(S_{TOPO},A_{TOPO}|\theta^{Q}\right){|}_{S=S_{t},A=\mu \left(S_{t}\right)}\nabla_{\theta^{\mu }}\mu \left(S|\theta^{\mu }\right){|}_{S_{TOPO,t}}$15:     Update the target networks parameters : $\theta^{Q^{\prime }}\leftarrow \tau \theta^{Q}+\left(1-\tau \right)\theta^{Q^{\prime }}$ $\theta^{\mu^{\prime }}\leftarrow \tau \theta^{\mu }+\left(1-\tau \right)\theta^{\mu^{\prime }}$16:   **end for**17:**end for**

In the proposed system, feedback is applied using the object function $y_{TOPO}$ in the actor network and the loss function $L_{TOPO}$ in the critic network. $L_{TOPO}$ is a loss function of the critic network, and the critic network updates in the direction of minimizing the loss. After the critic network is modified, the actor network updates the new policy using the modified value using the object function $y_{TOPO}$. In the general policy gradient of the system, the target value $y_{TOPO}$ for the target actor network can be calculated as follows:

(1) $$y_{TOPO,t}=R_{TOPO,t}+\gamma Q^{\prime }\left(S_{TOPO,t+1},\mu^{\prime }\left(S_{TOPO,t+1}|\theta^{\mu^{\prime }}\right)|\theta^{Q^{\prime }}\right).$$

After that, the calculated target value is updated through the following loss function:

(2) $$L_{TOPO}=\frac{1}{N}\sum_{t}^{}{\left(y_{TOPO,t}-Q\left(S_{TOPO,t},A_{TOPO,t}|\theta^{Q}\right)\right)}^{2}.$$

The actor network applies feedback from the critic network to maximize and update the objective function as follows:

(3) $$\nabla_{\theta^{\mu }}J\approx \frac{1}{N}\sum_{t}^{}\nabla_{A}Q\left(S,A|\theta^{Q}\right){|}_{S=S_{t},A=\mu \left(S_{t}\right)}\nabla_{\theta^{\mu }}\mu \left(S|\theta^{\mu }\right){|}_{S_{t}}.$$

Finally, the DDPG updates the parameters of the target neural network. Here, τ is a very small value, which determines the rate of reflection of the target network when updating:

(4) $$\theta^{Q^{\prime }}\leftarrow \tau \theta^{Q}+\left(1-\tau \right)\theta^{Q^{\prime }},$$

(5) $$\theta^{\mu^{\prime }}\leftarrow \tau \theta^{\mu }+\left(1-\tau \right)\theta^{\mu^{\prime }}.$$

### 3.4. Optimization Modeling

In RL, the optimization method commonly adopted for parameter learning is the ADAM. Typically, the step function for updating parameters iterates as much as the initial fixed value and then ends the episode. The number of step functions required for learning depends on the formation of the network topology. It is inefficient to use the same number of learning step functions for all network topologies. Therefore, the proposed system introduces an adaptive and variable learning processes that can flexibly reflect the modifying network topology. We use a modified step function to increase the learning rate of the entire reinforcement learning system. As the learning rate of the network topology increases, the time sensitivity of the system simultaneously increases. Algorithm 3 provides a detailed description of step function optimization.
**Algorithm 3** Step Function Optimization1:**while** exception occurred **do**2:   **if** $t>H_{done}$ **then**3:     **for** $t-H_{done}<k<t$ **do**4:        $F_{sum},F_{avg}\leftarrow 0$5:        $F_{sum}\leftarrow F_{sum}+R_{TOPO,k}$6:        $F_{avg}\leftarrow F_{sum}/H_{done}$7:        **if** $F_{avg}>H_{learn}$ **then**8:          $Epi_{done}=\mathrm{True}$9:        **else**10:          $Epi_{done}=\mathrm{False}$11:          $A_{size}\leftarrow A_{size}+A_{change}$12:        **end if**13:     **end for**14:   **end if**15:**end while**

As the learning is repeated within the episode, the step optimization function is executed when the number of step functions increases to more than the specific optimization hyperparameter $H_{done}$. In the step optimization function, the average of recent steps is obtained by the number of $H_{done}$ and compared with $H_{learn}$, which is a value close to the maximum reward result. If the average value $F_{avg}$ of the recent step is greater than $H_{learn}$, the learning of the corresponding episode is terminated. Otherwise, the step optimization function changes the size of the action and repeats the same process. The action size of the next step is adjusted to reflect the learning progress for each UAV network topology. The system runs iteratively and checks the reward of each step. If the average of recent step rewards remains high enough, the system evaluates that learning has converged and ends the episode. As a result, the proposed system optimizes learning by adjusting the size and number of steps used for training.

### 3.5. System Environment

The agents continually interact with the environment while performing learning, so it is important to construct an appropriate environment. As the episodes pass, UAVs move and form a new network topology. The proposed system initially forms the network topology with the number of sectors declared as hyperparameters. After that, the number of sectors for the optimal network topology is learned according to the changing location information by reflecting the learning results. In the proposed system, it is initially planned to train using 100 episodes of 1000 step functions. This subsection provides a detailed description of the state, action, and reward.

#### 3.5.1. State

The states used for learning are as follows:

(6) $$S_{TOPO,t}=\left[T_{hop,t},T_{power,t},T_{degree,t}\right]$$

The state is composed of three elements: hop, power consumption, and degree, which can best represent the UAV network topology. First, the hop means the average number of links each UAV has. Increasing the number of hops in a network topology means increasing the number of links in the network topology, which usually negatively affects the overall network topology. Second, power consumption measures the energy consumption of the link according to the distance between UAVs. Good energy efficiency can be obtained if the distance of the links constituting the network topology is efficiently planned. Third, the degree is the average of the number of UAVs that one UAV is connecting in one-hop. If it becomes too large, the overhead of the network increases. Since these three elements can represent various characteristics of the UAV network topology, they are suitable as a component of the state for the learning model.

#### 3.5.2. Action

In the proposed system, the number of sectors dividing the space around the UAV is used as an action for learning. As the number of sectors changes, the number of links that UAV connects to one-hop changes, so state components such as hop, power consumption, and degree are all affected. As a result, a change in action leads to a change in the next state, enabling smooth learning of the entire system.

#### 3.5.3. Reward

The rewards used for learning are as follows:

(7) $$R\left(t\right)=\left\{\begin{array}{cc} R_{disconnect}, & \mathrm{if}\;\mathrm{disconnection}\;\mathrm{occurs}\\ R_{out}, & \mathrm{if}\;A_{TOPO,t}>A_{border}\\ R_{TOPO,t}=H_{hop}*T_{hop,t}+ & \mathrm{otherwise}\\ \;\;\;\;\;\;\;\;H_{power}*T_{power,t}+H_{degree}*T_{degree,t} \end{array}\right.$$

Rewards are given in three cases. First, $R_{disconnect}$ is a reward given when UAVs in 3D space construct a network topology according to a given action, and unconnected UAVs occur. In this case, no reward is given and the system explores another action. The second is a reward given when the action exceeds $A_{border}$ due to increasing action. $A_{border}$ is set to 20 as a hyperparameter, and if the action value exceeds this, a negative reward is given for smooth learning because the calculated reward value is inappropriate for learning. The third is the case where the action is appropriately set so that learning can be performed by calculating the reward of the network topology. The reward is calculated as the weighted sum of the three hyperparameters of $H_{hop},H_{power}$ and $H_{degree}$ and the network components of $T_{hop,t},T_{power,t}$ and $T_{degree,t}$.

## 4. Performance Evaluation

We designed our proposed system and configured an environment that can perform the missions described in Section 3.1. We implemented the simulation using Python and measured the numerical data to plot the results using MATLAB. We used the OpenCV library to verify the network topology for various experimental conditions. We evaluated the proposed system by changing the formation shape, number of UAVs, and step function. Default value of each parameter is listed in Table 2.

### 4.1. Network Topology Control for Typical Formation

In order to check whether the learning of the proposed system proceeds smoothly, the training results of several typical deployments consisting of multiples UAVs were checked. Each typical structure considered the shape that the UAV fleet is likely to construct in a real environment. As a result, several typical UAV formations of 30 UAVs were deployed in the simulation space: sphere, cube and pyramid. The deployed UAVs hover by moving their position slightly, maintaining the first defined typical formation for 100 episodes. Because the position change of each UAV is small, learning converges quickly based on the proposed system. When the proposed system reaches a certain level of convergence, step optimization is applied to reduce the number of repeated step functions. The system confirmed that learning proceeds well in the network topology of each typical formation.

**Figure 3:**
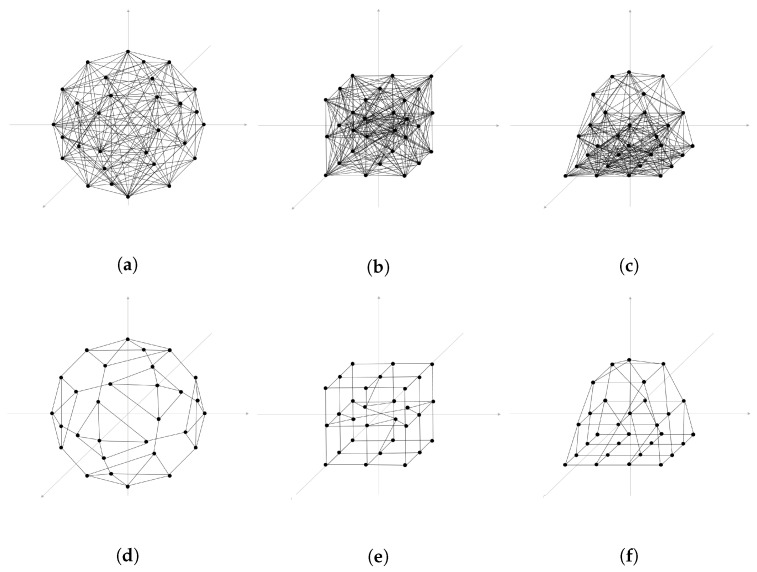
Network connectivity of typical UAV formation after training: (**a**) Full connection of Sphere. (**b**) Full connection of Cube. (**c**) Full connection of Pyramid. (**d**) Training result of Sphere. (**e**) Training result of Cube. (**f**) Training result of Pyramid.

Figure 3 compares the degree of connectivity before training ((a), (b), and (c)) and after training ((d), (e), and (f)). Figure 3a shows the initial network connectivity before training, with 30 UAVs are placed in a spherical shape in a three-dimensional space. In more detail, the spherical structure is a formation in which each UAV is placed almost similar distance from the center point of the sphere and is evenly spaced on the surface of the sphere. Similarly, Figure 3b shows the initial network connectivity before training, with 30 UAVs are placed in the form of a cube in a three-dimensional space. The cube structure uses a total of 26 UAVs to form the vertex of the cube and the center point of the cube surface, and 4 UAVs are placed in the middle layer inside the cube. Finally, Figure 3c shows the initial network connectivity before learning, with 30 UAVs placed in a pyramid shape within a three-dimensional space. The pyramid structure was considered a hierarchical form consisting of a total of four layers. The four layers are composed of 1, 4, 9, and 16 UAVs from the top, respectively, forming a pyramid shape. Before training, the network topology constructed by multiple UAVs consists of connections to all neighboring UAVs within the transmission range. The network topology without training shows a link connection state that significantly increases network overhead due to too many connections.

Figure 3d–f shows the learning results for the sphere, cube, and pyramid topology described above. After learning, the network topology has eliminated all unnecessary link connections compared to previous connection states. It was confirmed that as the episodes increased and learning progressed, the network topology remained in a stable formation.

**Figure 4:**
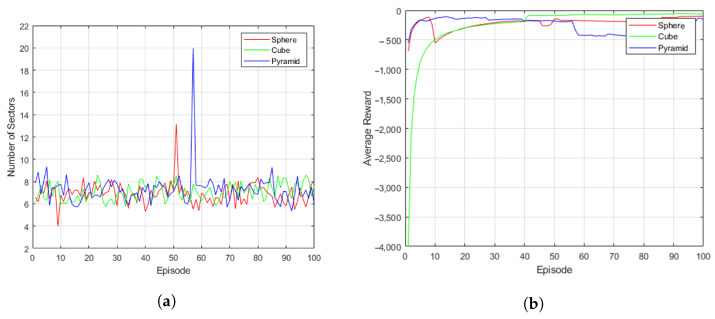
Simulation results of typical UAV formation (spheres, cubes, pyramids): (**a**) Subspace Change. (**b**) Episodic Reward.

Figure 4 shows the simulation result of typical UAV formations. Figure 4a shows the subspace change results for given UAV formations (sphere, cube, and pyramid). The simulation results show that all three typical formations show that the changes in the sector are quite subtle even though training proceeds with the increase in episodes. When the episode progressed about 50 times, it was confirmed that the system, which judged itself to have fallen into the local minima, went through the action search process for a while and then restored to a stable state.

Figure 4b shows the episodic reward for typical UAV formations. The episodic rewards had already converged to a near-optimal state before 10 episodes passed. Since the learning of the network topology was continuously output with high rewards, it could be observed that the episodic rewards were rather reduced when the exploration process for new actions was included.

### 4.2. Reinforcement Learning Results for Multiple Nodes

**Figure 5:**
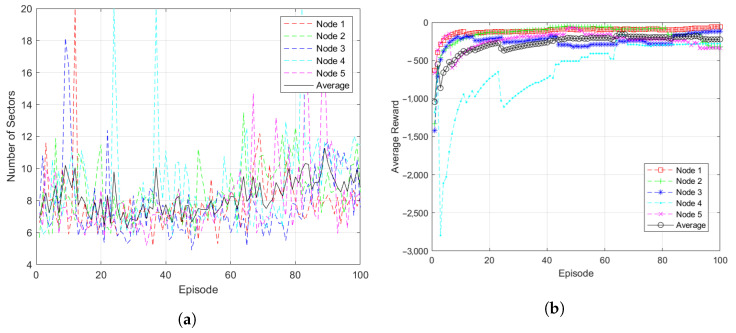
Simulation results of multiple nodes: (**a**) Subspace Change. (**b**) Episodic Reward.

Figure 5 compares the simulation results of 5 randomly selected nodes to check whether all nodes of the proposed system are trained. The five selected nodes were constituent nodes of a 30-node UAV network and their positions were changed at the start of each episode. The nodes divided the surrounding space using the position information and distance information of the surrounding nodes in each episode. Next, these nodes formed a network topology using the MST structure in the divided space.

Figure 5a shows the change in the number of sectors divided by each node as a result of learning. It can be seen that the average value of the sectors of the five nodes maintains between 7 and 10. It was confirmed that the position of each node was changed according to the episode, resulting in slight differences.

Figure 5b shows the episodic reward results of the five nodes. Although the degree of detailed learning varies depending on the position of the node, it was confirmed that a high reward value was obtained after about 60 episodes.

### 4.3. Network Topology Control for Arbitrary Formation

**Figure 6:**
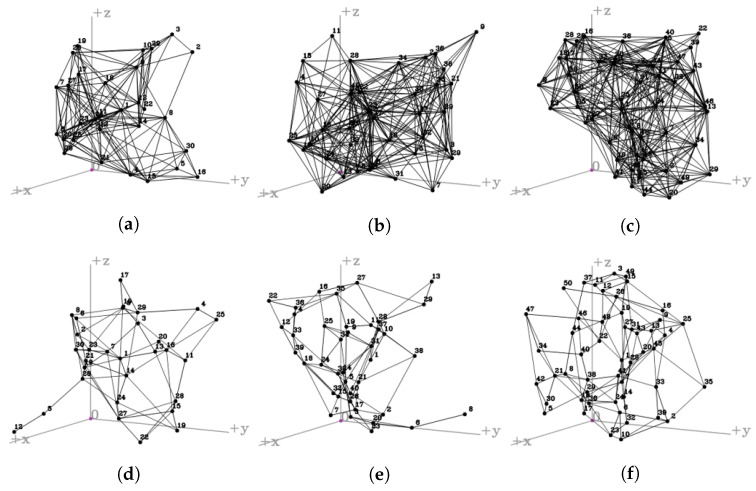
Network connectivity of arbitrary UAV formations after training: (**a**) Full connection of 30 UAVs. (**b**) Full connection of 40 UAVs. (**c**) Full connection of 50 UAVs. (**d**) Network topology control of 30 UAVs. (**e**) Network topology control of 40 UAVs. (**f**) Network topology control of 50 UAVs.

In order to verify that the proposed system works well in the real environment, we randomly placed UAVs in 3D space and observed the results. Furthermore, we have verified using various numbers of UAVs to evaluate the scalability of the proposed system. The simulations used 10, 20, 30, 40, and 50 UAVs, and the topology of the UAV fleet was updated every 100 episodes. Among the many simulation results, the network connectivity of the UAV fleet composed of 30, 40, and 50 UAVs was confirmed. Figure 6 shows the diffenrence in connectivity between before training ((a), (b), and (c)) and after training ((d), (e), and (f)).

Figure 6a–c shows the initial network connectivity before training, with 30, 40, and 50 UAVs are placed in arbitrary formation in a three-dimensional space, respectively. Figure 6a shows the network connectivity of UAV fleet composed of 30 UAVs are placed in arbitrary formation in a three-dimensional space. The 30 UAVs randomly placed in the virtual space updated information on the surrounding UAVs and connected links to construct the network topology. However, since the proposed system has not been applied, it is a topological state with many unnecessary links. Figure 6b,c shows the network connectivity of UAV fleet composed of 40 and 50 UAVs are placed in arbitrary formation in a three-dimensional space. Even though the number of UAVs increased to 40 or 50, the same pattern was shown at the beginning of the episode.

The proposed system constructed an efficient network topology by excluding unnecessary links as learning progressed. Training was performed consistently even though the number of UAVs constituting the network topology increased. These positive learning results were confirmed in Figure 6d–f. Figure 6d shows that a UAV fleet of 30 UAVs maintains an optimal topology. The number of links constituting the entire UAV network topology has been suitably reduced, resulting in improved network connectivity compared to before learning. Likewise, Figure 6e shows network connectivity of 40 UAVs after learning. As with other simulation results, it can be confirmed that all UAVs are connected to surrounding UAVs using at least one link without an isolated UAV, forming the entire network topology. Lastly, Figure 6f shows the network connectivity composed of 50 UAVs. Through the continuous simulation results for the proposed system, it was confirmed that even if the number of UAVs constituting the fleet increased, the learning for effective topology configuration was well performed.

**Figure 7:**
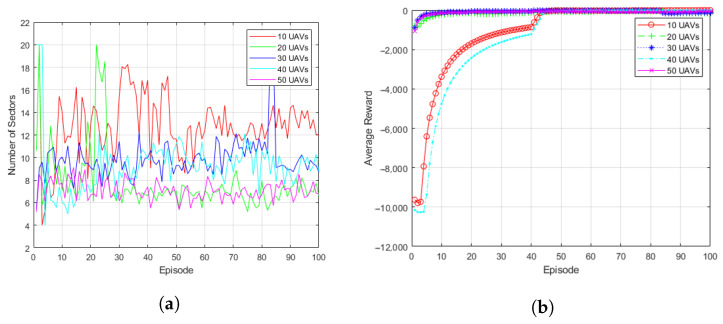
Simulation results of the arbitrary UAV formations (10, 20, 30, 40, 50 UAVs): (**a**) Subspace Change. (**b**) Episodic Reward.

Figure 7 shows the simulation result of arbitrary UAV formation. Figure 7a shows the subspace change results for given arbitrary UAV formations (10, 20, 30, 40, and 50 UAVs). The fleet composed of 10 UAVs conducted training to find the optimal topology while widely changing the subspace from 4 to 18. As a result, in a fleet of 10 UAVs, the proposed system learned that the topology constructed using about 12 subspaces is the optimal state. The number of subspaces is higher than that measured in a fleet with a larger number of UAVs. Since the number of UAVs in space is smaller than in other situations, the number of subspaces increased to connect links with as many UAVs as possible. Fleets composed of more than 20 UAVs showed almost similar learning results. Each fleet formed an optimal topology using about 6 to 10 subspaces.

Figure 7b shows the episodic reward for arbitrary UAV formations. It was confirmed that the reward convergence of the formation composed of more than 30 UAVs is performed before 10 episodes, whereas the reward convergence of the formation composed of 20 or less UAVs is performed at 40 episodes. The proposed system outputs the optimal network topology after a certain episode has passed in all verified situations.

A video of the simulation results is referred in [24]. This video contains the results of experiments conducted in Section 4.1 and Section 4.3. In the early part of the simulation, the learning results of the proposed system are visually shown for UAV formations randomly placed in 3D space using 30, 40, and 50 nodes. It can be seen that the optimal network topology is maintained even though the random formation formed by the UAV changes with the increase of episodes. The next part of the simulation shows the process of forming a typical UAV formation using 30 nodes and learning the most efficient network topology from that shape. In a typical UAV formation, 30 UAVs constituting a network sequentially change into a sphere-cube-pyramid shape. In the process of moving the formation, it can seen that the learning is not interrupted and the optimal topology is continuously maintained through the video.

### 4.4. Step Function Optimization

The verification related to the learning of the proposed system was conducted in both Section 4.1, Section 4.2 and Section 4.3. The simulations performed in the previous section are designed to allow learning to proceed for a long time by utilizing as many and sufficient number of step functions as possible for each episode, focusing on whether the proposed system learns or not. However, in order to optimize the network topology using UAVs, a fast learning rate that can reflect the high mobility of UAVs is essential. Therefore, two approaches for the number and size of steps for step function optimization were verified through simulation. By applying the changing step size and number of times, the change in the step function actually used for learning was verified.

**Figure 8:**
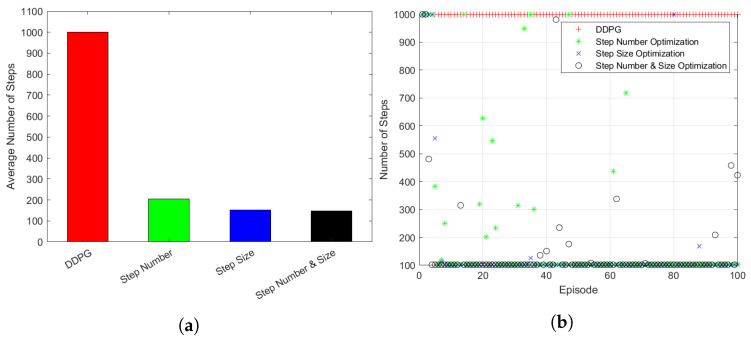
Change of steps after step optimization: (**a**) Average Number of Steps. (**b**) Number of Steps used in Episode.

Figure 8a shows the reduction in the actual number of steps used for learning. As a result of applying the variable step function algorithm, it was found that the number of steps used for training was reduced by 80% when the number of steps was changed and by 85% when the step size was changed. It was confirmed that when the number and size of steps were adjusted together, the number of steps was reduced by about 86%. Reducing the number of iterative step function executions directly means that the learning rate of the proposed system increases.

Figure 8b shows the number of step functions required for each episode in the learning process applying step optimization. The step optimization algorithm determines that learning has been completed to some extent, it ends the learning of the corresponding episode and moves on to the next episode. After a certain period of parameter learning, the overall number of step functions used for learning has decreased. Many step functions were used for learning in the early episodes of learning, but almost similar results could be obtained with a relatively small number of steps and a considerably shorter learning time after few episode progressed. The green and blue symbols in the graph each means the number of step functions used for step optimization when the number of step and the step size is changed. Subsequently, the black symbol indicates the number of step functions required for each episode when both the number and size of steps are changed for step optimization. As a result, it was confirmed that the optimization algorithm with variable steps helps to reduce the number of steps in an episode.

**Figure 9:**
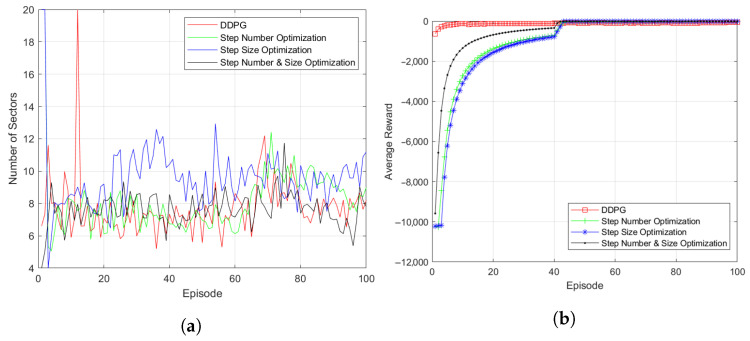
Simulation results of step optimization: (**a**) Subspace Change. (**b**) Episodic Reward.

Figure 9 compares the episodic reward results and sector change results of the existing algorithm with the step optimization results. Figure 9a shows the result of sector change simulation applying the step optimization method. The simulation used a formation in which 30 UAVs were randomly placed in the simulation space, and the entire system was learned through 100 episodes consisting of an initial 1000 step functions. Compared to the existing algorithm, the algorithm with step optimization showed similar sector change results, although the number of steps used for learning was decreased. In addition, more stable simulation results could be obtained from the algorithm integrating the number of steps and size adjustment.

Figure 9b shows that the step optimization algorithm using the variable number of steps obtains the episodic reward almost identical to the existing method. Even later in the learning, the use of step optimization algorithms showed higher episodic reward results despite using significantly fewer step functions for learning compared to existing DDPG algorithms. In addition, it was confirmed that higher rewards could be obtained faster when learned by integrating the number and size of steps.

### 4.5. Verification in Real Flight Scenarios

Prior to verifying the proposed system through actual UAV flight, we verified it in an AirSim environment that can fully reflect the actual environment. AirSim is a simulator developed by Microsoft for machine learning development. AirSim uses Unreal Engine 4 to visually reflect real-world environments in greater detail. In addition, this simulator provides an interface with Mavlink, and there are APIs for both pixhawk firmware such as PX4, python, and C++.

Furthermore, AirSim uses various sensors such as cameras and LiDAR to implement an environment that is almost identical to the real environment inside the simulator [25,26]. Accordingly, we validate our real UAV formation flight scenarios within AirSim. A video of the experiment can be found in [27].

**Figure 10:**
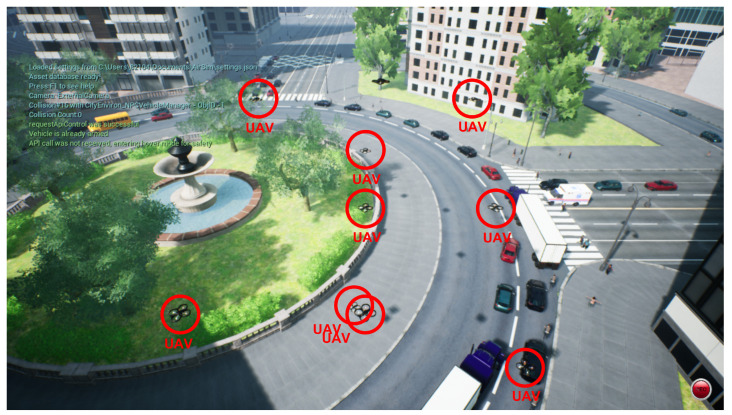
Simulation results of UAV topology control in AirSim.

Figure 10 shows the implementation of a scenario where a fleet of 10 UAVs is flying in an AirSim environment. In urban areas, UAV fleet can fly between buildings and fulfill a variety of roles, such as transporting logistics, flying performances, collecting weather data, controlling traffic conditions, and monitoring high-crime areas in the city. Efficient communication between UAVs is essential to fulfill these diverse roles. In the video, it was confirmed that the proposed system maintains the optimal topology for continuous UAV communication.

## 5. Conclusions

Recent advances in UAV technology have proposed the use of UAV fleets to provide wireless networks in environments where there is no network infrastructure. While this method has the advantage of providing a fast and inexpensive network, multi-hop connectivity and UAV control can cause scalability problems when trying to cover large areas. Therefore, the problem of efficiently controlling the network topology to form an UAV network is very important, and based on these observations, this paper proposes a RL-based topology control system for UAV networks.

The proposed system divides the surrounding space and configures the network topology for each subspace based on MST structure. In addition, the optimal network topology is constructed by solving the spatial partitioning problem through training using RL. Finally, by changing the number of steps of parameter learning, the time sensitivity and adaptability of the system are increased by interacting the changing environmental factors. Our research can be used to achieve efficient mission performance and energy efficiency in a network topology composed of multiple UAVs to perform various purposes.

We have several directions for future work. We will optimize the proposed system using more diverse learning algorithms. For deep learning-based learning algorithms or other reinforcement learning algorithm, more complex state, action, and reward designs can be developed. In addition, the optimal learning algorithm can be implemented by considering various learning algorithms in combination. We will implement an optimal learning algorithm by studying the reflection ratio of individual algorithms in the complex learning algorithm. Finally, further research can be conducted on the application of variable steps in learning. By applying variable steps to learning through more complex modeling, faster training speed will be achieved.

## Figures and Tables

**Table 1:** Variables used in proposed system.

Notation	Description	Notation	Description
$P_{UAV}$	Position information of UAVs	*t*	Number of steps per time period
$D_{UAV}$	Distance information of UAVs	$Step_{max}$	Maximum number of steps
$G_{UAV}$	Geometry information of UAVs	$y_{TOPO}$	The target value of RL
$T_{hop}$	Average number of hops for the network topology	$L_{TOPO}$	The loss function of RL
$T_{power}$	Average energy consumption of topology links	$\theta^{Q}$	Critic network parameter
$T_{degree}$	Average degree of the all network topology nodes	$\theta^{\mu }$	Actor network parameter
$S_{TOPO}$	State value of the topology	*B*	Replay buffer value of DDPG
$A_{TOPO}$	Action value used in RL	*N*	Noise used in RL
$A_{size}$	Step size of action	τ	Very small value for parameter update
$A_{change}$	Variable step size of action	$F_{sum}$	Sum of the reward results of the last steps
$R_{TOPO}$	Reward value used in RL	$F_{avg}$	Average of the reward results of the last steps
$Epi_{max}$	Total number of episodes	$H_{done}$	Hyperparameters that control the number of steps used for step optimization
$Epi_{done}$	T/F value to end the episode	$H_{learn}$	Hyperparameters of results close to the upper limit of rewards

**Table 2:** Simulation parameters.

Item	Value	Item	Value
Space size	1000 m × 1000 m × 1000 m	Critic network learning rate	0.002
Number of UAVs	10, 20, 30, 40, 50	Actor network learning rate	0.001
Maximum transmission power	20 dBm	Total episodes	100
Frequency band	2.5 GHz	discount factor gamma	0.99
Antenna gain	2.5 dBi	τ	0.005
Receive signal threshold	−70 dBm	Buffer size	(50,000, 64)

## Abbreviations

The following abbreviations are used in this manuscript:

DDPG	Deep Deterministic Policy Gradient
RL	Reinforcement Learning
ADAM	ADAptive Moment Estimation
MST	Minimum Spanning Tree
